# Critical role of accompanying authorized surrogates among the multifactorial determinants of in-hospital delays in patients with acute stroke

**DOI:** 10.3389/fneur.2026.1757307

**Published:** 2026-05-08

**Authors:** Haoyu Wang, Wei Ni, Yao Chen, Haifang Yu, Yarong He

**Affiliations:** 1 Emergency Department, West China Hospital, Sichuan University, Chengdu, Sichuan, China; 2 Emergency Department, Chengdu Shangjin Nanfu Hospital, Chengdu, Sichuan, China

**Keywords:** accompanying authorized surrogate, acute stroke, emergency treatment time, informed consent, in-hospital delay, outcomes

## Abstract

**Background:**

Despite the widespread establishment of stroke centers, delays in pre-hospital and in-hospital care persist. To date, no studies have investigated the role of accompanying authorized surrogates in early emergency care timelines and the outcomes of acute stroke. This study aimed to analyze the influence of types of surrogate decision-maker on critical time intervals and early clinical outcomes.

**Methods:**

This retrospective cohort study was conducted in patients with acute stroke or those with suspected stroke admitted to a stroke center. The correlation between the type of accompanying authorized surrogate at patient admission and both early in-hospital emergency timelines [medical order, computed tomography (CT) imaging and emergency treatment times] and early prognosis (neurological outcome, incidence of adverse events) was analyzed using statistical methods, including univariate and multivariate regression analysis.

**Results:**

A total of 508 patients were managed via the stroke fast-track; among these, 414 were diagnosed with acute stroke. The type of accompanying authorized surrogate significantly influenced all in-hospital treatment time metrics. Patients without a surrogate decision-maker experienced significantly longer orders, CT imaging, and emergency treatment times than those with a surrogate (*p* < 0.05). Notably, patients whose colleague or friend was the surrogate decision-maker had the shortest in-hospital emergency treatment time (*p* < 0.05). Patients who sought medical care alone had the shortest length of hospital stay, lowest incidence of adverse events, and best neurofunctional outcomes.

**Conclusion:**

Early in-hospital delays in patients with acute stroke are multifactorial. The presence and type of accompanying authorized surrogates have a considerable effect on in-hospital emergency treatment time for acute stroke. However, the type of accompanying authorized surrogate showed a weak correlation with early prognosis in patients with acute stroke.

## 1 Introduction

Acute stroke can manifest as either acute ischemic stroke or hemorrhagic stroke and is a global health burden and leading cause of disability and mortality (1–3). Despite divergent treatment strategies and prognosis, for both subtypes share a “universal imperative”: the therapeutic window for successful clinical intervention depends entirely on the prompt diagnosis and immediate treatment (4–8).

While pre-hospital delays are well-documented as a significant factor of poor patient outcomes (9, 10), patients also play a pivotal role in mitigating or exacerbating in-hospital delays. This role is particularly evident in situations involving delays or the refusal of informed consent (9, 11). In China, the model of medical decision-making presents a distinct contrast to the Western paradigm; informed consent is a legally mandated process where family members, rather than the patient, often serve as the primary decision-making makers (12). This arrangement is especially pronounced in cases of acute stroke, in which a substantial proportion of patients present with cognitive or neurological impairments that limit their capacity for autonomous consent (13, 14). To date, no studies have explored the role of authorized surrogates in early emergency care timelines and the outcomes of acute stroke. Hence, we hypothesized that for patients with acute stroke presenting to a stroke center, the absence of a legally authorized surrogate decision-maker, as well as the specific type of surrogate present, significantly influences key in-hospital emergency treatment timelines and early clinical outcomes. The objective of this study was to establish a basis for graded communication strategies for stroke center staff and to inform improvements in emergency pathways, facilitating the timely and effective management of patients with acute stroke.

## 2 Methods

### 2.1 Research design

This study retrospectively evaluated consecutive patients with acute stroke or those suspected acute stroke at a stroke center emergency department from January 1, 2023, to August 31, 2025, to assess the influence of surrogate decision-maker type on key early in-hospital emergency care intervals.

### 2.2 Study participants

The study included consecutive patients aged 14 years or older who presented to our stroke center between January 1, 2023, and August 31, 2025, with a diagnosis of suspected stroke and symptom onset within 24 h. Patients were excluded from the analysis based on the following criteria: missing critical clinical data, transfer from another hospital where the initial diagnosis or management was initiated, and failure to undergo cranial computed tomography (CT) imaging.

### 2.3 Data collection

Data were collected retrospectively by reviewing Hospital Information System (HIS) and paper-based medical records. For each patient presenting to our stroke center between January 1, 2023, and August 31, 2025, the following clinical data were extracted: sex, age (years), comorbidities, disease course, time of presentation, medical order time, CT imaging time, emergency treatment time, presence or absence of a surrogate decision-maker, and the relationship between the surrogate and the patient. The prognostic indicators assessed were hospital stay, neurological outcomes [Glasgow-Pittsburgh Cerebral Performance Category (CPC)], with an unfavorable outcome (CPC score ≥ 3) and a favorable outcome group (CPC score ≤ 2). Documented adverse events included endotracheal intubation, intensive care unit (ICU) admission, and death.

### 2.4 Definition of time metrics

The disease course was defined as the onset-to-door time, which refers to the interval between symptom onset and patient arrival at the stroke center. Medical order time was defined as the interval from patient arrival (The door of an emergency center) to issuance of the first medical order by a stroke center physician, as recorded in the HIS. The CT imaging time was defined as the interval from patient arrival to completion of the CT localizer scan. The emergency treatment time was defined as the interval from patient arrival to the completion of hospital admission.

### 2.5 Grouping criteria

According to the guidelines and the standards for the establishment of stroke centers, patients were categorized into adequate and inadequate groups based on whether the order time was completed within 10 min. Similarly, patients were classified into CT-adequate and CT-inadequate groups based on whether the CT imaging was completed within 25 min. The types of accompanying surrogates were divided into groups representing those who arrived alone, spouses, immediate family, relatives, and others (such as colleagues and friends).

### 2.6 Outcomes

The primary outcomes were CT imaging findings, medical orders and emergency treatment times. The secondary outcomes were length of hospital stay, adverse events and favorable neurological outcome with CPC ≤ 2.

### 2.7 Statistical analysis

Continuous variables with a normal distribution are presented as means ± standard deviations and were compared via Student’s *t*-test. Continuous variables with nonnormal distribution are expressed as medians and interquartile ranges. Comparisons between two groups were performed using Mann–Whitney U test and comparisons among multiple groups using Kruskal–Wallis test. Categorical data are summarized as frequencies and percentages, and group differences were assessed using the chi-square test. Multivariable logistic regression analysis was used to identify the factors influencing the timeline of early emergency care. All statistical analyses were performed using SPSS 27.0 (IBM Corporation) and ORIGIN 2025 (OriginLab Corporation) software. Statistical significance was set at *p* < 0.05.

## 3 Results

**Figure 1:**
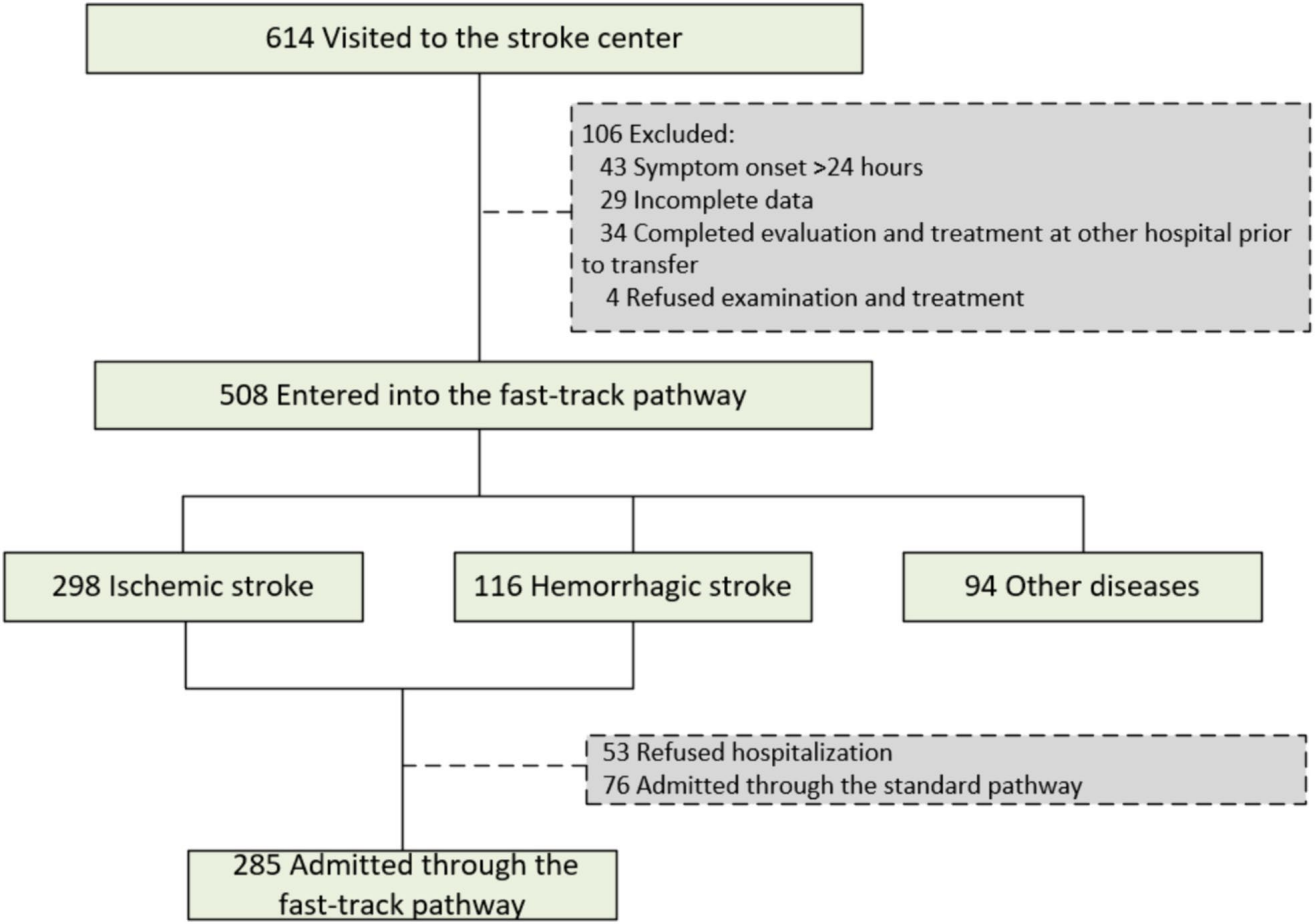
Flow of patients through the study. During a 32-month study period, 614 patients were triaged to the emergency stroke center. Among these, 508 patients presented within 24 h of symptom onset and were managed through the emergency fast-track pathway. Acute stroke was confirmed in 414 patients, among whom 285 were immediately admitted for further treatment in accordance with the fast-track pathway admission criteria.

Between January 1, 2023, and August 31, 2025, a total of 614 patients were initially evaluated at our stroke center. Of these, 508 were enrolled in the fast-track stroke protocol for further management. The final diagnoses of the 508 patients included 298 (58.7%) cases of acute ischemic stroke and 116 (22.8%) of hemorrhagic stroke (Figure 1). A total of 414 stroke patients met the criteria for hospital admission. However, 53 (12.8%) patients refused hospitalization. Among the remaining patients, 285 (68.8% of the total stroke cohort) were admitted directly via fast-track, whereas 76 (18.4%) were admitted through common pathways.

When assessing whether the medical order time and CT imaging time met the required standards, this study revealed between-group differences in terms of visit time, disease course, diagnosis, admission status after the visit, and the type of accompanying authorized surrogate (*p* < 0.05) (Table 1). Stroke patients with daytime visits and longer disease duration were more likely to experience delays in order and CT imaging time. Stroke patients without an accompanying authorized surrogate were more prone to order delays, whereas having a colleague or friend as the medical consent provider was associated with a lower risk of delays in both order and CT imaging times. Multivariate logistic regression analysis revealed that visit time, disease course, diagnosis, and admission situation were factors influencing whether the medical order and CT imaging times met the required standards (*p* < 0.05). The type of accompanying authorized surrogate was identified as a factor affecting whether the CT imaging time met the required standards (*p* < 0.001).

**Table 1:** Demographic characteristics of patients with acute stroke.

Variables		Medical order time (minutes)				CT imaging time (minutes)			
		Adequate group (*n* = 416)	Inadequate group (*n* = 92)	P*	P**	Adequate group (*n* = 416)	Inadequate group (*n* = 92)	P*	P**
Sex									
	Male	273 (65.6%)	56 (60.9%)	0.388	–	156 (69.6%)	173 (60.9%)	0.041	0.124
Age (years)		65.202 ± 13.909	65.511 ± 15.877	0.851	–	64.527 ± 14.181	65.834 ± 14.338	0.306	–
	≥60	153 (36.8%)	33 (35.9%)	0.905	–	83 (37.1%)	103 (36.3%)	0.855	–
Visit time									
	Daytime	242 (58.2%)	65 (70.7%)	0.034	0.045	113 (50.4%)	194 (68.3%)	<0.001	<0.001
History of diseases									
	Yes	291 (70.0%)	62 (67.4%)	0.619	–	153 (68.3%)	200 (70.4%)	0.607	–
History of stroke									
	Yes	51 (12.3%)	10 (10.9%)	0.860	–	26 (11.6%)	35 (12.3%)	0.805	–
Disease course		3.941 ± 5.192	8.232 ± 7.611	<0.001	0.049	3.390 ± 4.044	5.465 ± 6.904	<0.001	0.013
	≤4.5 h	306 (73.6%)	40 (43.5%)	<0.001	0.778	169 (75.4%)	177 (62.3%)	0.002	0.672
Diagnosis									
	Hemorrhagic stroke	107 (25.7%)	9 (9.8%)			66 (29.5%)	50 (17.6%)		
	Ischemic stroke	249 (59.9%)	49 (53.3%)	<0.001	0.001	132 (58.9%)	166 (58.5%)	<0.001	0.099
	Other	60 (14.4%)	34 (36.2%)			26 (11.6%)	68 (23.9%)		
Type of surrogate									
	Alone	4 (1.0%)	6 (60%)			3 (1.3%)	7 (2.5%)		
	Spouse	133 (32.0%)	23 (25.0%)			49 (21.9%)	107 (37.7%)		
	Family	239 (57.0%)	57 (62.0%)	0.006	0.825	144 (64.3%)	152 (53.5%)	<0.001	<0.001
	Relatives	18 (4.3%)	2 (2.2%)			12 (5.4%)	8 (2.8%)		
	Others	22 (5.3%)	4 (4.3%)			16 (7.1%)	10 (3.5%)		
Admission situation									
	Refused	73 (17.5%)	31 (33.7%)			28 (12.5%)	76 (26.8%)		
	Common pathways	68 (16.3%)	36 (39.1%)	<0.001	<0.001	34 (15.2%)	70 (24.6%)	<0.001	<0.001
	Fast-track	275 (66.1%)	25 (27.2%)			162 (72.3%)	138 (48.6%)		

P*: *P*-value from univariate analysis; P**: *P*-value from multivariate logistic regression analysis.

**Figure 2:**
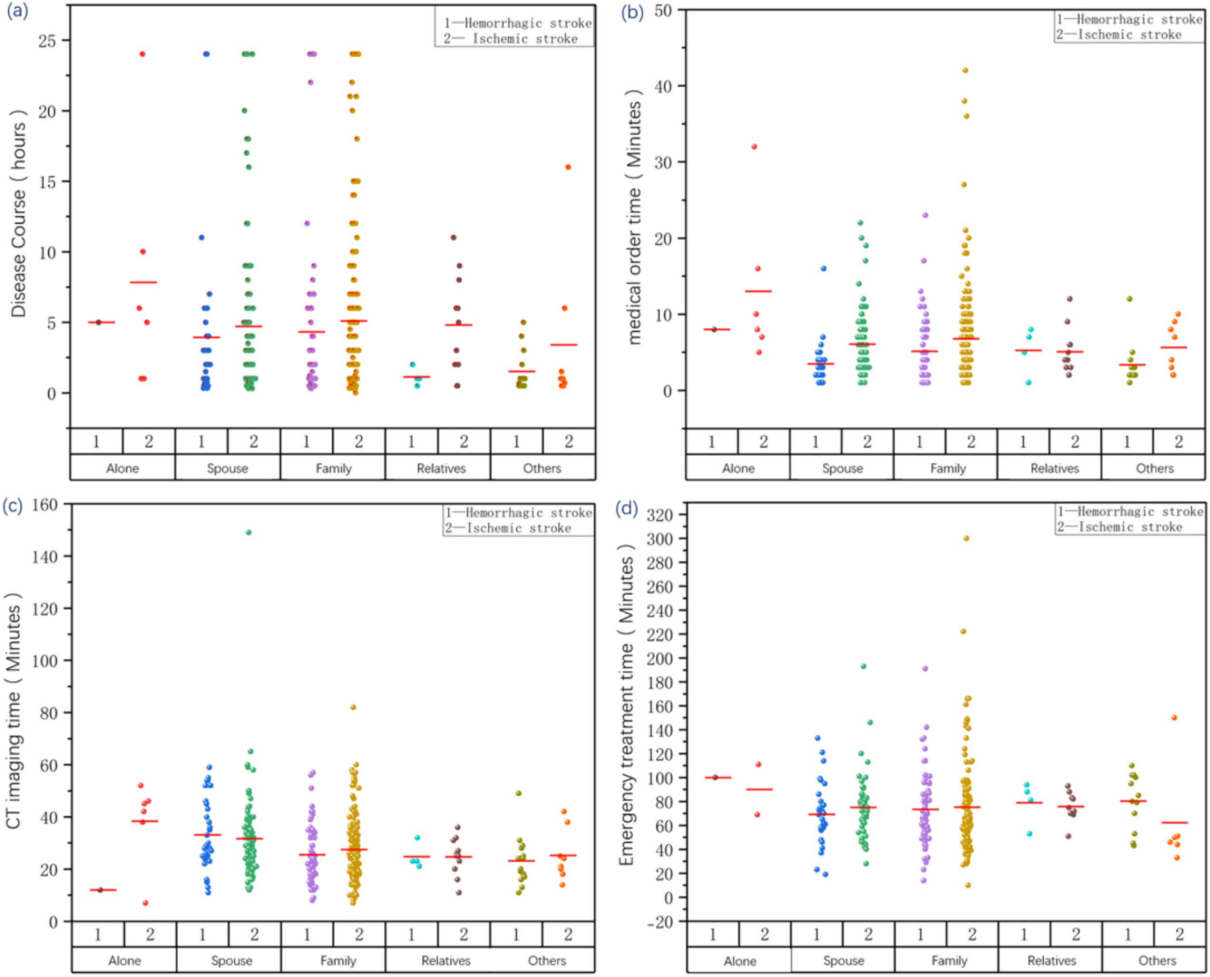
Scatter plot with bars depicting the relationship between types of surrogate decision-makers and treatment times in patients with acute stroke. Panel **(a)** shows the distribution of disease course across different types of accompanying authorized surrogate among 414 patients with acute ischemic and hemorrhagic stroke, indicating no significant difference (*p* > 0.05). Panel **(b)** presents the distribution of medical order time across different types of accompanying authorized surrogate among 414 patients with acute ischemic and hemorrhagic stroke, demonstrating a significant difference (*p* < 0.001). Panel **(c)** illustrates the distribution of CT imaging time across different types of accompanying authorized surrogate among 414 patients with acute ischemic and hemorrhagic stroke, also showing a statistically significant difference (*p* < 0.001). Panel **(d)** depicts the distribution of emergency treatment time across different types of accompanying authorized surrogate among 285 patients with acute ischemic and hemorrhagic stroke admitted via the fast-track pathway, with no statistically significant difference between groups (*p* > 0.05). CT, computed tomography.

As shown in Figure 2, the type of accompanying authorized surrogate had a significant effect on both the medical order time and CT imaging time in patients with acute stroke (*p* = 0.002 and *p* < 0.001, respectively). However, no significant association between disease course and emergency treatment time was observed (*p* = 0.170 and *p* = 0.883, respectively).

Further analysis of demographic characteristics revealed that older patients with acute stroke tended to be accompanied by their spouses or family members and were more likely to seek medical care during daytime hours (*p* < 0.05) (Table 2). The disease course in stroke patients without an accompanying authorized surrogate (11.333 ± 10.969 h) was significantly longer than that in patients with an accompanying authorized surrogate (*p* < 0.05). When a friend or colleague served as the accompanying authorized surrogate, the patient’s disease course was the shortest (1.196 ± 1.147 h). The CT imaging time for patients with acute stroke who sought medical care alone or with their spouse as the accompanying authorized surrogate was significantly longer than that for patients with other types of authorized surrogates (*p* = 0.005). No significant differences in order or emergency treatment times were observed among the different types of accompanying authorized surrogates. However, patients with acute stroke who sought medical care alone exhibited the longest order and emergency treatment times, whereas those who were accompanied by a friend or colleague had the shortest order and emergency treatment times. No significant differences (*p* ≥ 0.05) were observed in hospital stay, neurological outcomes, or the incidence of adverse events between the groups in terms of the type of accompanying authorized surrogate. However, patients who sought medical care alone had the shortest length of hospital stay, lowest incidence of adverse events, and best neurofunctional outcomes.

**Table 2:** Demographic characteristics and outcomes among different accompanying authorized surrogate groups for patients admitted via the fast-track.

Variables	Alone (*n* = 3)	Spouse (*n* = 77)	Family (*n* = 174)	Relatives (*n* = 13)	Others (*n* = 18)	*P*
Sex						
Male	3 (100%)	55 (71.4%)	108 (62.1%)	11 (84.6%)	17 (94.4%)	0.017
Age (years)	52.667 ± 23.861	58.429 ± 10.255	71.540 ± 10.936	50.077 ± 13.444	41.944 ± 10.333	<0.001
≥60	1 (33.3%)	27 (35.1%)	146 (83.9%)	2 (15.4%)	0 (0%)	<0.001
Visit time						
Daytime	3 (100%)	45 (58.4%)	103 (59.2%)	4 (30.8%)	15 (83.3%)	0.030
History of diseases						
Yes	3 (100%)	51 (66.2%)	121 (69.5%)	7 (53.8%)	9 (50%)	0.251
History of stroke						
Yes	0 (0.0%)	8 (10.4%)	23 (13.2%)	2 (15.4%)	2 (0.0%)	0.478
Disease course (h)	11.333 ± 10.969	3.039 ± 4.075	3.594 ± 4.653	3.346 ± 3.356	1.196 ± 1.147	0.005
≤4.5 h	0 (0.0%)	64 (83.1%)	131 (75.3%)	9 (69.2%)	17 (94.4%)	0.003
Diagnosis						
Hemorrhagic stroke	1 (33.3%)	29 (37.7%)	57 (32.8%)	4 (30.8%)	12 (66.7%)	0.079
Ischemic stroke	2 (66.7%)	48 (62.3%)	117 (67.2%)	9 (69.2%)	6 (33.3%)	
Medication order time (minutes)	7.667 ± 0.577	4.273 ± 2.698	5.201 ± 3.645	4.769 ± 2.421	3.833 ± 2.915	0.088
CT imaging time (minutes)	33.333 ± 18.583	30.130 ± 11.925	25.287 ± 9.915	24.846 ± 6.986	23.167 ± 9.569	0.005
Emergency treatment time (minutes)	93.333 ± 21.779	72.909 ± 27.667	74.672 ± 37.468	76.846 ± 13.716	74.333 ± 31.311	0.883
Hospital stay (days)	8.667 ± 7.371	12.883 ± 11.293	12.753 ± 10.162	15.000 ± 13.620	10.611 ± 5.932	0.767
Adverse events						
Endotracheal intubation	0 (0%)	14 (18.2%)	19 (10.9%)	4 (30.8%)	4 (22.2%)	0.145
ICU admission	0 (0%)	15 (19.5%)	20 (11.5%)	5 (38.5%)	4 (22.2%)	0.050
Death	0 (0%)	9 (11.7%)	18 (10.3%)	3 (23.1%)	2 (11.1%)	0.669
Overall incidence	0 (0%)	17 (22.1%)	25 (14.4%)	4 (30.8%)	4 (22.2%)	0.310
Neurological outcome						
CPC score	1.333 ± 0.577	2.377 ± 1.328	2.362 ± 1.291	2.385 ± 1.660	2.500 ± 1.383	0.731
Favorable neurological outcome	3 (100%)	41 (53.2%)	98 (56.3%)	8 (61.5%)	9 (50%)	0.555

**Figure 3:**
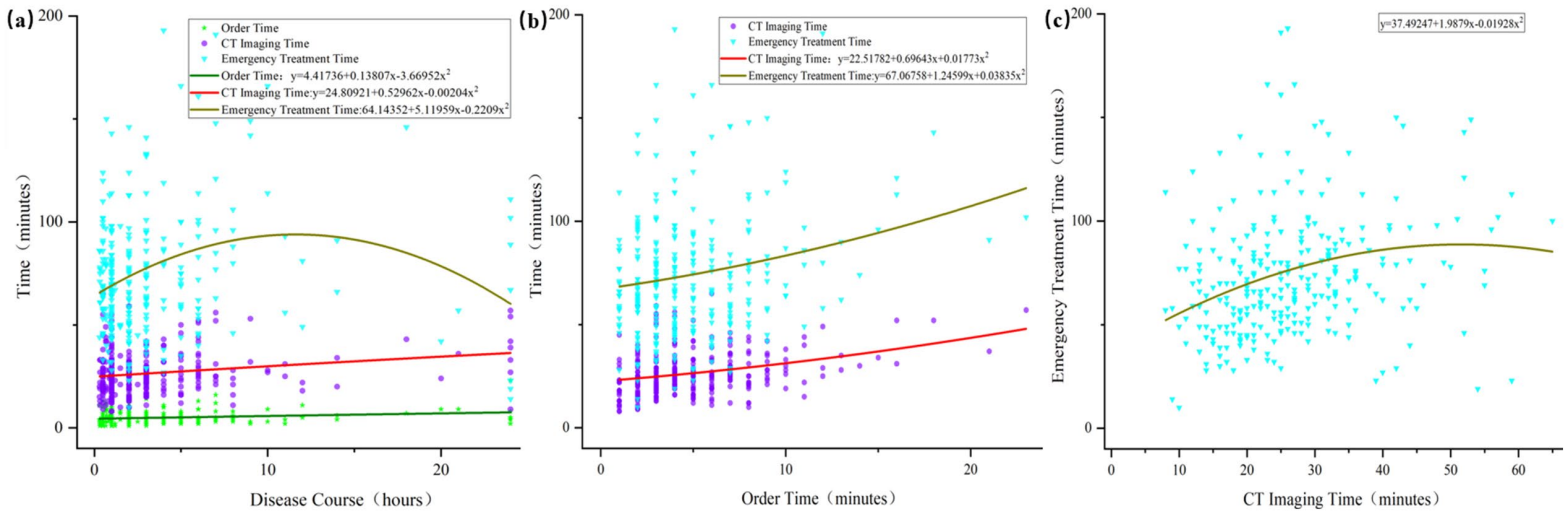
Modeling of key early time intervals in patients with acute stroke. Correlation analysis among disease course, medical order time, CT imaging time, and emergency treatment time in 285 patients with acute stroke. Panel **(a)** shows that disease course exhibits no significant correlation with medical order time, a weak positive correlation with CT imaging time, and a significant correlation with emergency treatment time within a defined interval. Panel **(b)** demonstrates that medical order time is positively correlated with both CT imaging time and emergency treatment time. Panel **(c)** indicates a strong positive correlation between CT imaging time and emergency treatment time. CT, computed tomography.

Finally, based on the aforementioned influence of the accompanying authorized surrogates on the patient’s early care timeline, we analyzed the interactive effects among these timeline variables (Figure 3) and found a sequential linear correlation between disease course, medical order time, CT imaging time, and emergency treatment time.

## 4 Discussion

The clinical axiom “Time is brain, CT is brain” underscores the critical necessity of minimizing every possible delay in diagnosis and treatment (15, 16). While the establishment of stroke centers has clearly improved patient outcomes (17, 18), the diagnostic and treatment steps such as neuroimaging, reperfusion therapy, and surgical interventions require informed consent which remains a substantial hurdle. Hence, expediting the consent process is vital to minimize in-hospital delays (19). The time required for informed consent is highly variable and influenced by numerous factors, including stroke severity, health literacy, financial constraints, patient decision-making capacity, and family dynamics (20, 21). The novelty of this study lies in its pioneering investigation of how accompanying authorized surrogates influence the early emergency care timeline and outcomes of acute stroke.

Our study suggests that early in-hospital delays in patients with acute stroke are multifactorial. While informed consent and decision-making behavior significantly affect this delay, they do not affect early prognosis. The presence or absence of a surrogate decision-maker and the specific type of surrogate significantly affect key in-hospital emergency treatment times for patients with acute stroke. The absence of a surrogate was associated with markedly prolonged emergency treatment time. Intriguingly, patients whose friends or colleagues were the surrogate decision- makers experienced the shortest delays. This finding may be explained by the complex and urgent nature of communication required in acute stroke, which encompasses discussions on the diagnosis, imaging findings, treatment options, prognosis, and costs. When patients present with cognitive or neurological impairments, which is common in acute stroke, the physician’s assessment and consent processes can be severely hindered, leading to significant treatment delays. Therefore, alternative consent models should be considered to address these challenges. For instance, digital informed consent tools may help streamline the process, particularly for vulnerable populations such as older individuals, unaccompanied patients, or those with limited literacy or writing ability (22). Furthermore, transitioning from written to verbal consent for time-sensitive interventions could further reduce in-hospital delays (23). The implementation of such alternatives, however, requires robust support from legal frameworks, institutional policies, and ethical guidelines (13, 14). Therefore, under the current paradigm, healthcare teams must continue to employ all available means to secure timely consent from legally authorized surrogates to ensure the best possible outcomes for patients with acute stroke.

Stroke can occur in various settings, and the social context at onset significantly influences subsequent help-seeking behaviors. When a stroke occurs in the presence of friends or colleagues, individuals who are motivated to mitigate adverse outcomes, typically facilitate prompt emergency calls, seek immediate medical attention, and contact the family. Consequently, such patients generally receive timely and proactive medical care. This trend is more frequently observed in younger patients, who tends to exhibit a more positive preference for aggressive treatment (24). Contrastingly, patients who experience a stroke in isolation often face significant delays. These delays are influenced by a complex interplay of objective variables, such as advanced age, sex, and regional socioeconomic status, and subjective factors, such as lack of symptom recognition (25). Furthermore, the absence of a surrogate decision-maker at presentation introduces further complexities. This can lead to difficulties in obtaining an accurate medical history and necessitates repeated confirmation or consultations with bystanders and paramedics to formulate a viable a treatment plan. Further, obtaining informed consent is challenging when a patient lacks decision-making capacity and arrives unaccompanied. Healthcare providers must navigate complex legal and procedural pathways that may involve police intervention to locate family members, inevitably causing critical treatment delays. Furthermore, even when a surrogate is present, the inherent characteristics of the stroke play an important role. For example, older patients and their spouses, who are often of similar ages, may require more time to comprehend consent forms and discuss the financial implications. Frequently, decisions regarding the treatment of older patients are deferred until after telephone discussions with their adult children, who are the primary decision-makers. This phenomenon is pervasive in China and affects both prehospital and in-hospital care pathways.

Pre-hospital delays remain a significant global concern. Stroke severity and specific patient characteristics are well-documented factors that influence the disease course (24, 25). Our findings are consistent with this observation, demonstrating a median prehospital delay exceeding 4 h, with approximately one-third of the patients arriving beyond the 4.5-h window. Notably, a history of stroke was associated with significantly shorter delays, suggesting improved symptom recognition. Within the hospital setting, patients with ischemic stroke and those who ultimately refused hospitalization experienced prolonged emergency treatment time. This evidence collectively underscores the substantial impact of patient- and system-related factors on pre-hospital and in-hospital delays. The barriers to timely care are multifaceted. One study delineated a three-tiered model of prehospital delay: the first delay stems from the failure to recognize stroke severity (38.4%), the second from presenting to an under-equipped medical facility (20%), and the third from insufficient financial capacity (10.8%) (20). Despite the growing global burden of stroke, the current preventive strategies remain insufficient (26, 27). While public awareness remains a critical issue, knowledge of the critical “golden hour” for treatment remains alarmingly low at 4% (28). To bridge this gap, targeted interventions such as developing stroke education applications tailored to older individuals and their families and enhancing the diagnostic capabilities of community hospitals needs to be implemented (29, 30).

An important question arising from our data is whether patient-related factors are the primary contributors to pre-hospital and in-hospital treatment delays. Intriguingly, our study revealed that patients who presented at night had a shorter duration of in-hospital emergency treatment (*p* < 0.05). We speculate that this may be partly attributable to the healthcare resource allocation. During daytime hours, radiology departments are responsible for performing CT scans not only for emergency cases, but also for outpatients and inpatients. Only a minority of hospitals are equipped with CT scanners dedicated exclusively to emergency use. To address this challenge, stroke centers have established fast-track protocols aimed at minimizing the CT imaging time for patients with stroke. However, this issue has not been fully resolved. In most hospitals, optimizing strategies to further shorten the CT imaging time for patients with stroke presenting during the day remains a priority. This finding suggests that when hospital traffic is reduced and resource allocation is streamlined, the system can achieve higher throughput efficiency.

While the establishment of stroke centers has substantially reduced in-hospital delays and mitigated the impact of hospital system factors on stroke care efficiency, significant scope remains for improvement from the healthcare system perspective. For example, the implementation of pre-hospital telemedicine via video transmission for consultations can further streamline in-hospital workflows and reduce delays (31). The creation of mobile stroke units, which initiate diagnostic workup and treatment before hospital arrival, has been shown to markedly shorten CT imaging and overall treatment times (32). Although patient-related factors can directly contribute to treatment delays, the role of healthcare providers in the stroke care pathway remains indispensable, particularly in communication and recommendation (33, 34). Effective communication between patients and physicians is widely recognized as the primary method for overcoming cultural and knowledge barriers. Therefore, enhancing physicians’ cultural competence and communication skills can help mitigate bias, improve patient experience, and facilitate the timely acquisition of informed consent (35–37). While communication in emergency settings differs significantly from that in elective situations, patients and their surrogates in acute scenarios express a need for detailed information and opportunities to ask questions (38). The management of acute stroke imposes persistent time pressure on physicians and patients/surrogates, necessitating efficient and effective communication strategies. Current evidence suggests that the use of visual aids, animations, and other visualization technologies may assist physicians in helping surrogates reach a decision more efficiently (39, 40). Importantly, throughout this process, regardless of the presence or type of surrogate, stroke center physicians must act prudently, respecting the autonomy of the patient and their surrogates and avoiding legal liabilities (39, 41).

Our analysis revealed no significant differences (*p* ≥ 0.05) in hospital stay, neurological outcomes, or the incidence of adverse events across the various surrogate categories. This intriguing outcome suggests that while the type of surrogate impacts the time needed to obtain consent, it may not fundamentally alter the early clinical trajectory once admission is secured, warranting further investigations. Since critical decisions concerning surgical, interventional, or thrombolytic therapy are finalized during emergency care, the post-admission phase does not have pressing time constraints. This provides a window for comprehensive discussion among patients and their families as well as detailed consultations with physicians. This scenario underscores the distinct nature of elective decision-making processes compared with those in critical, time-sensitive situations. Importantly, the methodology and design of the present study may have introduced potential biases, thereby influencing the observed results. The relatively small sample size in certain subgroups may have attenuated the statistical power, potentially obscuring true differences in outcomes. Furthermore, the primary outcome was assessed using the CPC scale at hospital discharge, which may not have fully captured the dynamic nature of neurological recovery or reflected functional improvements achieved during subsequent rehabilitation. To address these limitations, future research with larger, more robust sample sizes and extended follow-up periods is warranted to validate our preliminary findings.

The study identified a linear correlation among the medical order, CT imaging, and overall emergency treatment times. This finding suggests that minimizing delays at each individual stage may contribute to a reduction in total emergency management time, thereby facilitating more timely intervention for stroke patients. Emphasis on stage-specific time targets and increased attention to the role of patient–family dynamics may prove beneficial in adhering to the time constraints recommended by stroke management guidelines.

This study examines the impact of accompanying authorized surrogates on early emergency timelines and short-term outcomes in patients with acute stroke, with findings that may inform optimization of stroke pathways and the informed consent process. However, this study has certain limitations. First, the retrospective design precludes causal inference and limits our ability to determine the impact of specific patient-related factors, such as financial capacity, cognitive function, and risk aversion, on in-hospital delays. Second, although multiple individuals may participate in the patient’s care, final informed consent is typically obtained from a single primary surrogate, potentially oversimplifying complex decision-making dynamics. Future prospective studies should address these limitations.

## 5 Conclusion

Our findings demonstrate that early in-hospital delays in acute stroke are multifactorial, and that the presence and type of authorized surrogates significantly influence in-hospital emergency time metrics. However, the type of accompanying authorized surrogate shows a weak correlation with early prognosis in patients with acute stroke. Furthermore, although stroke centers are widely established globally, CT imaging delays remain a contributing factor to in-hospital delays, indicating that in-hospital resource allocation and emergency response protocols require further optimization. Finally, enhancing stroke education and public awareness campaigns targeting the older populations represents a promising strategy for reducing pre-hospital delays.

## Data Availability

The original contributions presented in the study are included in the article/supplementary material, further inquiries can be directed to the corresponding authors.
